# Registered Nurses’ Experiences of End-of-Life Care in Nursing Homes of South Korea: A Qualitative Study

**DOI:** 10.3390/healthcare10112213

**Published:** 2022-11-04

**Authors:** Soo-Jung Chang

**Affiliations:** Department of Nursing, Gangneung-Wonju National University, 150, Namwon-ro, Wonju 26403, Korea; sjchang@gwnu.ac.kr; Tel.: +82-33-760-8645

**Keywords:** end-of-life care, nurses, nursing homes, interview, perception, qualitative research

## Abstract

This study aimed to qualitatively describe how registered nurses (RNs) experienced and perceived end-of-life (EOL) care for older residents in South Korean nursing homes. The participants included 11 nurses with experience of providing EOL care for older residents in six nursing homes. Data were collected through one-on-one in-depth interviews using semi-structured questions from 27 December 2019 to 22 October 2020. Around 1–2 interviews were conducted for each participant, and each interview lasted between 40 min and two hours. The interview data were analyzed using qualitative content analysis. Eight sub-themes were identified and abstracted under three themes: (a) “feeling fulfilled for doing my best until the last day,” (b) “defensive coping due to legal and institutional limitations,” and (c) “requirements for effective EOL care.” This study suggests that there are many challenges and issues regarding EOL care at nursing homes. In order to provide effective EOL care to older residents, the foundation of the EOL care system, as well as skills training, should be strengthened. Furthermore, securing qualified nursing manpower and achieving institutional improvement by reducing obstacles are warranted.

## 1. Introduction

Interest in dying well, an important component of quality of life, has increased worldwide [1]. The association between quality of life and dying is stronger in the older age group than in any other demographic. One explanation for this is that loss is particularly experienced during old age through various forms, such as decreased function and productivity, retirement, and death, especially through the bereavement of a spouse. Consequently, death has greater impact on the overall life of older adults [2].

Given the rapid increase in the older population and life expectancy in South Korea, there is a consensus that a dignified human life should include not only the present life but also a good death [3]. Consequently, the 2016 Hospice-Palliative Care and Life-Sustaining Treatment Decision-Making Act, known as the “Dying Well Law,” has been in effect since 2017 [1]. However, this Act is still in its preliminary stages; it focuses on a single stage and a type of hospitalization-oriented specialized palliative care for patients with four major terminal diseases, including cancer. The Act has certain limitations in meeting the needs of various patients in the process of dying, as well as their families [1]. Furthermore, since institutional support for end-of-life (EOL) care regarding older adults in nursing homes is not connected with South Korea’s long-term care insurance (LTCI) system, the effect of the Act has been limited.

The LTCI was established in 2008 to promote health and stable life conditions in old age and relieve the burden of care-taking families. There were 1754 nursing homes at the end of 2008 [4], which rapidly increased to 5988 by the end of 2021 [5]. Consequently, more than 150,000 older residents currently live in Korean nursing homes [6]. Results from tracking and analyzing the data of 131,802 individuals aged 65 and above, who died in 2018, indicated that the average length of time spent in nursing homes during the 10 years before their death was 707 days [7]. Furthermore, nursing home residents’ medical expenses in the month before their death were more than three times higher than those one year prior; 31.8% of older residents received life-sustaining treatment during the month preceding their death [8]. Similarly, EOL treatment and dying are unavoidable processes in caring for older residents, and nursing homes are often the settings where these occur.

During EOL care for older residents, discussions about assessing and reflecting on residents’ and their families’ needs, and what kind of medical, psychological, and spiritual support should be provided, are required. However, policies and academic interest regarding nursing homes have mainly focused on the quality of services, health promotion of the residents, and reduction of burden for the families [9,10,11,12], while issues concerning EOL care for older residents have not received much attention.

In Korean nursing homes, those taking direct care of older residents are registered nurses (RNs), certified nursing assistants (CNAs), and certified caregivers. Among them, RNs play a major care role as the only medical professionals performing close observation of residents, pain management, infection control and wound care, managing CNAs and certified caregivers, communicating with medical staff, and running quality management programs [13]. However, because of the employee placement policy that allows CNAs to take the position of RNs, nurse staffing levels are so low that 6 out of 10 nursing homes lack RNs [14]. The Institute of Medicine (IOM) in the United States (US) recommends that all nursing homes should provide EOL care aimed at palliative support to respect the needs of individuals during EOL, and improve their quality of life [15]. Achieving these goals requires identifying challenges or aspects to be improved in EOL care through the experience of RNs, who are the closest to older residents during their EOL stage, and are largely responsible for their EOL care in nursing homes [16]. It is also important to consider the cultural background and institutional conditions related to providing EOL care. Unlike Europe and the US, where extensive research on the EOL care provided by RNs in nursing homes has been conducted [16,17,18,19], in South Korea, this topic has been studied mainly in hospitals [20,21,22], with few studies on nursing homes. To fill this gap, our study aimed to describe the EOL care experiences of RNs in Korean nursing homes. The study is expected to contribute to establishing basic data and improving the EOL care system in nursing homes.

## 2. Materials and Methods

### 2.1. Study Design and Research Question

This was a qualitative study conducted through in-depth interviews and qualitative content analysis to describe the experiences of RNs about EOL care in nursing homes. The following research question was investigated: “What are the experiences of RNs toward EOL care for nursing home residents?”.

### 2.2. Definition of EOL in This study

Since it is difficult to predict death due to the varied death trajectories in older adults [23], this study defined EOL as the period during which death is imminent, and the patient is in a state of dependence on others for daily life [24].

### 2.3. Setting and Participants

In this study, RNs from six nursing homes in one metropolitan city and four provinces of South Korea were recruited and contacted through purposive and snowball sampling. All six nursing homes were operated by private owners and three of them were operated by RNs; two of the homes had 100 or more beds, two had 30–99 beds, and two had 9–29 beds. The inclusion criteria required RNs currently working at a nursing home for more than three months, and having experience in providing EOL care for older residents. In South Korea, the number of RNs per facility is 0.28 in accordance with the legally recommended ratio of RN (or CNA) to residents, which is 1:25 [6]. Considering that RNs provided direct nursing care regardless of their position in the nursing homes, managers or directors were included as well.

### 2.4. Ethical Considerations

This study was performed after ethical approval from the Institutional Review Board of Gangneung-Wonju National University (No. GWNUIRB-2019-25-1). The researcher explained the research purpose, methods, and procedures to the facility management by e-mail or phone, and obtained support for participant recruitment and data collection. Before each interview, all participants were informed about the purpose, methods, procedure of the study, voluntary participation, the possibility of discontinuance or withdrawal at any time, and that all data would be treated anonymously and confidentially. Written informed consent was obtained from all participants before data collection.

### 2.5. Data Collection

Data were collected by the author (an RN and Ph.D. with extensive experience in qualitative study) through one-on-one, in-depth interviews using semi-structured questions from 27 December 2019 to 22 October 2020. Face-to-face interviews were performed with 10 participants in a confidential and separate room at the nursing homes, or a place of the participants’ convenience; an online video interview using Zoom was conducted with one participant. Interview questions were composed as one central question with seven sub-questions revolving around the person (e.g., the situation of dying residents, communication or interaction with people related to residents), time (e.g., caring process during and after death of residents), environment (e.g., policies of nursing homes, difference between nursing homes, and other settings), and facilitators or barriers of EOL care (Table 1).

Around 1–2 interviews were conducted for each participant, including a follow-up interview in case unclear content was found through the transcription, with each interview lasting for 40 min to two hours. All interviews were recorded and transcribed verbatim by the author.

### 2.6. Data Analysis

The interviews were analyzed by the author using a qualitative content analysis method [25]. The qualitative content analysis includes open-ended starting points with a person-centered approach and suggests a comprehensive philosophical background to understand specific phenomena from participants’ points of view [26]. Moreover, data from interviews and observations are collected from interactions between researchers and participants, which are based on communication theory [25]. In this study, after reading the interview data repeatedly and grasping the overall meaning, the meaning units were extracted by charting meaningful words, sentences, and paragraphs related to the research question. After naming the extracted meaning units as codes through a compression process that reduces the content while maintaining the core, similar codes were grouped and derived into sub-themes by comparing similarities and differences between the content, and these were re-integrated and abstracted into themes.

### 2.7. Trustworthiness

To assure the credibility of the research results as suggested by Lincoln and Guba [27], participants were encouraged to freely express their experiences through open-ended questions, without intervention. The recorded material was transcribed and checked to ensure that the transcriptions were complete. Unclear or confusing contents were reconfirmed and supplemented by telephone or follow-up interviews. At each stage, the similarities and differences between data belonging to codes, sub-themes, or themes were comparatively analyzed. Furthermore, for conceptual validation of the interpretations, feedback was taken from two professors with extensive experience in qualitative research regarding the classification of sub-themes and themes. Similarly, two participants were asked to review the results. To assure transferability, participants were recruited from various nursing homes considering their number of beds and regions. To assure auditability, the research methods and procedures were described in as much detail as possible. Furthermore, to assure confirmability, an effort was made to exclude researcher biases and prejudices through reflection at each interview and analysis.

## 3. Results

Eleven RNs participated in the in-depth interviews; all of them were women with a median age of 50 years (range 27–59 years), median total clinical career of 15 years (range 4–30 years), and median working career in nursing homes of six years (range 0.7–15 years). Of these, nine participants were married. Furthermore, five participants had an associate degree, four had a bachelor’s degree, and two had a master’s degree. The positions they held were of staff nurse (*n* = 4), nurse team manager (*n* = 4), and director (*n* = 3) (Table 2).

The RNs’ experiences regarding EOL care were integrated into eight sub-themes, which were abstracted under three themes: (a) “feeling fulfilled for doing my best until the last day,” (b) “defensive coping due to legal and institutional limitations,” and (c) “requirements for effective EOL care” (Table 3).

### 3.1. Theme: “Feeling Fulfilled for Doing My Best until the Last Day”

#### 3.1.1. Sub-Theme: “Fulfilling the Duty for the Older Residents and Families”

Participants made efforts to fulfill their duties toward the older residents, whose physical functions were gradually deteriorating. They considered EOL care for the older residents as a natural duty and responsibility of RNs, and emphasized and encouraged RNs to directly take care of the older residents instead of entrusting certified caregivers with the tasks.

*“I firmly tell nurses—‘Nurses, I do not think it is right to give all the nursing work to certified caregivers. That is our responsibility. I hope you do not forget our roles.’”*

(Participant C)

The participants were monitoring and providing more careful and intensive care than usual for the older residents at the EOL stage to identify and address their basic needs. The participants stated that they felt further encouraged to do their best in the job when they noticed the older residents showing more comfortable facial expressions and becoming stable through their care.

*“We view the older residents as very special. We often check on them. Nurses are very careful about everything as well. Most importantly, we strive to provide emotional support. When all the staff members solely focus on the older residents, they show outstanding improvements in their psychological status, by initially displaying comfortable facial expressions.”*

(Participant E)

As such, the best EOL care provided by participants for the older residents is based on the intention that they recognize older residents as respect-worthy human beings with dignity, rather than just dying patients, and the hope to protect themselves from feelings of remorse or guilt.

*“In this nursing home, they are just the older adults rather than patients. I think it would be helpful for the older residents if I humanly consider their lives, which is also my duty, so I think that if I fulfill my duty, the older residents would be happy. … I am supposed to see many deaths of the older residents. If I am hurt whenever I see them, I would not be able to continue to work.”*

(Participant F)

Furthermore, participants tried their best in their duties toward the families of the older residents. In situations where the older residents showed steady physical deterioration without improvements, the participants explained their conditions to the families so that they could decide between life-sustaining treatment and preparation for death, and provided appropriate medical information, considering the position of the older resident and their families. Moreover, they believed that their roles involved supporting the families of older residents to navigate through the dying process.

*“I think that it is our role to ask families about the plan for the funeral of the older residents at the time of their deaths and to help them make decisions for life-sustaining treatments. I think it is also important for nurses to give accurate information to the families about the dying process of the older resident.”*

(Participant F)

#### 3.1.2. Sub-Theme: “Feeling Proud to Be Able to Do Something for the Older Residents at the EOL Stage”

The participants felt proud and comfortable when they did their best for the older residents at their EOL stage.

*“I felt proud because I could wash their bodies, close their eyes, and stick by them without being scared and experiencing a sense of denial before the older residents passed away… It does not feel too bad that I am a person who could do something for them until the last moment.”*

(Participant G)

### 3.2. Theme: “Defensive Coping due to Legal and Institutional Limitations”

#### 3.2.1. Sub-Theme: “The Burden of Responsibility as the Sole Healthcare Professional in the Nursing Home Setting”

The participants felt the burden of responsibility as the sole healthcare professional in the nursing homes. In situations where they had to make medical decisions on their own owing to a change in the older resident’s condition, based on their limited knowledge and medical equipment, participants were not confident about their judgment, which made them feel anxious.

*“Practically, there is no doctor here, and certified caregivers do not know about medical treatments. So, I try to make all decisions on my own if the conditions of the older residents become worse during my duty. Therefore, I have too much work to do, and have the biggest responsibility. I need to monitor things from A to Z and make decisions on treatments. So, I always wonder if those decisions were made correctly and I honestly feel anxious.”*

(Participant J)

They felt stressed due to the families relying on them for the decision of medical treatments regarding older residents. Moreover, they felt confused and troubled due to the different responses of families to pre-emptive actions in emergency situations.

*“In case of emergency, we call the family and transport the older resident to a hospital. If the state of the older resident improves in the hospital, the family sometimes strongly blames us for saying that it was too seriously managed even when it was a light condition. In one case, when an older resident could not eat, a Levin tube insertion led to a dispute among the family members, for instance between the older resident’s daughter and daughter-in-law. A family asked me, ‘Do you keep the one who is supposed to die still surviving by inserting a Levin tube?’ When I said, ‘The doctor said a Levin tube needs to be inserted,’ the family asked me, ‘Is it necessary? Should I trust you, manager?’ Then, what should I say? They also said, ‘Please make a decision.’ Subsequently, I made the decision, which was very stressful.”*

(Participant C)

As such, when the responsibility was focused on one RN alone, they felt remorseful that the death of the older resident might be their fault, or that they should have made more efforts in the care even though the death was inevitable.

*“When there was a sudden death of an older resident, I hated myself. I thought, ‘What is this? I should have cared more extensively… I should have asked about the condition more specifically.’ At that time, I had all sorts of skepticism, and then I felt remorseful, thinking that ‘it was not all that I saw.’”*

(Participant I)

#### 3.2.2. Sub-Theme: “Legal Restrictions to Nurses’ Roles and Limited Authority”

Due to the absence of a detailed manual, the procedure for EOL care or the communication process with the family for Do-Not-Resuscitate (DNR) was on an ad hoc basis, depending on the facility’s condition and RN’s capacity.

*“We did not make a protocol. The nurse and I have a discussion, it is case-by-case, based on the situation.”*

(Participant K)

The roles and legal authority of RNs were limited in nursing homes, which acted as a serious obstacle in providing proper EOL care.

*“I would like to give more oxygen, or hope to extend the life of the older residents by a few more days by giving the fluid. However, I cannot do it in the nursing home arbitrarily, as you know, due to the legal issue… I cannot do in the nursing homes even for controlling the pain. It is still illegal to provide hospice care in nursing homes. I think that is a big obstacle.”*

(Participant F)

The participants were unable to have a close communication regarding EOL care with on-call doctors in nursing homes or receive practical help from them, due to their limited roles (e.g., visiting a nursing home every two weeks to examine residents and prescribe medications), as well as passive and defensive attitudes. As for the older residents who had decided to face death in the nursing home, nurses could only provide the lowest level of basic treatments. Thus, they had to passively watch the pain of the older residents, which made them heavy-hearted.

*“No matter how easily families talked about the death previously, it is not easy from the standpoint of an RN. Basically, I can only give oxygen and measure oxygen saturation, and blood pressure… That is all I can do.”*

(Participant D)

#### 3.2.3. Sub-Theme: “Higher Priority to Operating a Facility Than Supporting Older Residents”

Participants mentioned that many established nursing homes vanished every year due to legal and institutional limitations, and that there was stiff competition among nursing homes, wherein they had to prioritize facility profits over their own beliefs. Furthermore, they said that some facilities took in only those older adults who could be managed, or actively transported those who were near the EOL stage to a hospital after cardio-pulmonary resuscitation (CPR), even though the older resident had submitted a DNR Consent Form. This was done to prevent any negative effects of the death on the other residents, and avoid any legal disputes with the families. Additionally, they had trouble listening to and solving complaints from certified caregivers. They behaved in a defensive manner considering that they might be blamed for residents’ deaths, and placed respect for the older residents at the EOL stage as a second priority.

*“Nursing homes spring up everywhere, as they just comprise a business. If they want to provide proper EOL care, it would not be profitable. That can cause the last moments to be so lonely rather than treating a human as a human.”*

(Participant H)

*“When a resident dies in a nursing home, a postmortem examination report is issued, not a death certificate. If so, an autopsy will be required to determine the cause of death, and legal disputes may arise in the process. So, I think that ‘an older resident’s death may be our responsibility’ rather than ‘I will take good care of him or her comfortably’. Sometimes, it’s sad. A resident passed away a few days ago. His guardian did not want him to go to the hospital, but as soon as I thought it could be a legal issue, I had no choice but to send him to the hospital while continuing CPR.”*

(Participant I)

### 3.3. Theme: “Requirements for Effective EOL Care”

#### 3.3.1. Sub-Theme: “Building Trust with the Older Resident’s Family, Nursing Home Employees, and On-Call Doctors as a Buffer Preventing Legal Issues”

The participants posited that trust-building was important to provide quality EOL care. They mentioned the need for collaboration and sharing care-related goals between the head of the facility and middle managers, who have leadership skills and a sense of responsibility, and staff members. Further, trust should be established with family members through frequent communication, and effective collaborative relationships with on-call doctors should be maintained. According to the participants, if those requirements were achieved, EOL care would be smooth, consequently reducing any legal burden and stress.

*“I think that the trust between families and the facility is very important. Trust is the method that can avoid making an issue a (legal) problem…I think that how well we know about the older person in advance, and how much we communicate with the families is important.”*

(Participant K)

#### 3.3.2. Sub-Theme: “EOL Care Education for Nursing Staffs, Residents, and Their Families”

Given that the accumulation of knowledge and experiences would decrease anxiety regarding care for the dying residents, as well as caregivers’ attitudes, participants wanted practical education for RNs and certified caregivers to cope with emergency situations, as well as hospice education. Additionally, they felt that death preparation education was also required for the older residents and their families.

*“We need education. The education changed my mental attitude and the attitudes that I had previously. I felt that this education was so important… Because it made me feel something and later, I could remember and apply the important principles during the sharing process with staff members, even though my basic life attitude cannot be changed.”*

(Participant C)

#### 3.3.3. Sub-Theme: “Institutional Support for EOL Care in Nursing Homes ”

The participants mentioned that legal and institutional support was required to provide effective EOL care in nursing homes. To correctly judge the changes in conditions and risk signs among older residents, they emphasized that professional RNs should be urgently recruited, and they should be institutionally trained.

*“When we are absent, CNAs do our work. But they are not aware of ‘risk signs’ of the older residents. Also, nurses without clinical experiences cannot judge the conditions of the older residents. I think we need more manpower for good EOL care, as that will be more helpful.”*

(Participant E)

They hoped for legal support to facilitate a comfortable death at the residential places for the older adults.

*“Institutionally, if the older residents are allowed to die here comfortably and we can announce deaths, they do not have to move. Am I wrong? If such a system is prepared, I think older adults would face death more comfortably.”*

(Participant J)

They also mentioned that the negative perception of the public toward nursing homes could be improved.

*“I believe that dying in nursing homes would be much better for the older adults. As long as families do not raise legal issues, we can do it much better. Since decision-making is in the hands of families and they do make a big difference, I think it is important to encourage families to take it more comfortably. I think the National Health Insurance Service should help in changing people’s perceptions.”*

(Participant E)

## 4. Discussion

This study found that the experiences of RNs regarding EOL care in nursing homes involved three themes, namely “feeling fulfilled for doing my best until the last day,” “defensive coping due to legal and institutional limitations,” and “requirements for effective EOL care.” The results suggest that there are many challenges and issues to improve in EOL care at nursing homes.

The study participants respected the older residents at the EOL stage as dignified humans and took efforts to fulfill their duties toward the older residents and their families. This result was consistent with previous findings [20,24] reporting that RNs provided holistic nursing care to older residents at the EOL stage, and satisfied their demands. According to the participants, one of their essential roles was to help families prepare for and make decisions regarding EOL care, and stay near the older residents at their EOL stage. This reflects the Korean perception of peaceful death surrounded by family as a good death [2,20,24]. This is somewhat different from the perceptions of other Asian countries, including “sudden death without suffering” in Japan, or Singapore’s emphasis on “death at home” [28]. The notion of EOL in South Korea began with the tradition of children holding the hands and feet of older adults and watching them during their EOL stage; it now means staying with the older adult and sharing the EOL moment with family and relatives. This is considered children’s essential duty [29]. Therefore, helping the family share the EOL of the older resident not only takes into account quality of life, but also implies the nurse’s crucial responsibility in helping the family fulfill their final duty regarding EOL care. When the participants did their best to care for the older residents at the EOL stage and their families until the end, they felt a sense of accomplishment. This was consistent with previous studies reporting that the psychological burden toward EOL could be reduced, depending on one’s efforts [21,30]. This could be viewed as a positive outcome from the accumulation of experiences, as RNs realized that an ideal EOL care involved perceiving death as part of the journey of life and fulfilling one’s duties in such processes [30].

This study revealed several legal and institutional barriers to providing high-quality EOL care at nursing homes. The characteristics of the older residents at the EOL stage, the shortage of RNs, and families of older residents leaving the responsibility of decision-making entirely to RNs were a huge burden for the participants, who were the only healthcare professionals at the nursing homes. The older residents presented greater medical demands as they approached EOL [31]. Moreover, the progression of EOL cannot be generalized as it is highly diverse and non-specific, and such ambiguity makes treatment at the right time or post-treatment care much more challenging [28,31]. Although medical judgment and appropriate measures for older residents at the EOL stage are frequently needed [32], RNs are absent in several nursing homes due to regulations in South Korea allowing the replacement of RNs with CNAs [33]. Reportedly, the number of long-term residents assigned to one RN has increased from 79 in 2008, to 224 in 2018. Hence, even when there are RNs, their duties and responsibilities have increased, making it difficult for them to prevent the worsening of resident health, or provide appropriate care [34]. Moreover, there is a risk of additional problems occurring at the time of emergency [32].

The heavy burden of decision-making assigned to participants by residents’ families was consistent with previous study results [32]. This presents the reality that an increasing number of families are shifting the responsibility of taking care of their elderly onto the public system, unlike the original intention of the LTCI system, which was to lessen the burden of family members and strengthen respect and care within the family [32]. Findings of participant guilt and regret at the unavoidable death of residents were also affirmed in other studies [30,32]. This can be explained by the “mirroring experience,” whereby participants linked residents’ dignity with their own. In this way, failing to provide high-quality EOL care can lead to burnout, with participants viewing themselves as undignified or demoralized [35].

When providing EOL care, participants had to rely entirely on their experiences and abilities without any standardized manual or practical help, due to the limited role and passive behavior of on-call doctors. This was in line with previous findings reporting that RNs provide experience-based care rather than evidence-based palliative care to patients with severe dementia [16,22]; furthermore, the study results highlighted a shortage of mentors for consultation and poor collaboration with physicians [17,18].

Taking into account legal and institutional limitations involving meticulous administrative procedures for EOL care by nursing homes, participants tended to be defensive in their care against their beliefs. These results were not prominent in previous studies on the experience of hospital nurses’ EOL care for elderly patients [21,22,36] and show how RNs may protect themselves in Korean nursing homes without doctors. Frequent hospital transfers at the EOL stage after life-sustaining treatment reflects the reality of South Korea, where hospital deaths account for 80.4% of deaths among dementia patients aged 60 years or above [37]. This far exceeds the hospital death rates of older residents from nursing homes, accounting for more than half in Japan [38], and one-third in Germany [19]. Hospitalization at the EOL stage cannot fulfill the EOL care goal of “improving comfort” [38] and is instead associated with adverse health effects [19]. Previous studies reported that hospitalization experiences of older residents with “Do Not Hospitalize” and DNR indications during their last year of life significantly decreased [38,39], and hospitalization rates dropped after initiatives to reduce hospitalizations were taken [40]. Hence, this finding shows that institutional improvement for promoting EOL care in nursing homes is needed.

According to participants, maintaining trusting relationships between residents’ families, employees, and on-call doctors is crucial for reducing burden and stress during EOL decision-making. It is similar to observations in previous studies [16,18,41,42,43]. When family members understand the progressive decline of the older resident and are mentally prepared, they can better serve their role as an advocate [41]. Therefore, communication and collaboration with residents’ families contribute to a reduction in hospital deaths and decision-making pressure [16,41,42]. Communication and collaboration with frontline staff allow an early understanding of the EOL condition of older residents, increasing the possibility of using hospice care [43]. Furthermore, to provide high-quality EOL care for older residents, a collaboration of various experts working as a team is critical [18,24,41,44]. Keeping these issues in mind, programs that provide training in communication for sharing prognostic information and decision-making, as well as for strengthening teamwork need to be developed and offered to nursing home employees.

The participants emphasized the need for practical emergency-handling education, hospice education, and education on preparing older residents and their families for death. This is one of the issues commonly emphasized in EOL care studies worldwide [16,17,18,19,20]. RNs who provide EOL care with minimum or no prior education experience negative emotions including fear, depression, and guilt; however, competency and professional knowledge can reduce the stress resulting from such heavy duties [45]. An improvement in understanding and better attitudes toward the different processes of EOL, as well as the reinforcement of self-confidence and self-efficacy in nursing home employees through education, can enhance the quality of EOL care [16,17,18,19]. However, only 20% of Korean nursing colleges offer an independent curriculum for EOL care, and students who have completed this course account for only 5.1% [46]. Furthermore, although EOL skills and competency are highly required in nursing homes [44], continuing education for RNs is almost absent in Korean nursing homes. A few scoping reviews, which mainly reviewed prior studies from Europe and North America, have also indicated that nursing staff may lack sufficient competencies, sensitive awareness, and personal attitudes to deal with complex EOL care needs of nursing home residents [18,44]. Therefore, there is an urgent need to promote institutional measures that include EOL care practical education for RNs in nursing homes.

An EOL preparatory education for older residents and their families plays a central role for the transition from care centered on life-sustaining treatment, which burdens the older residents, to palliative care [41]. Furthermore, helping older residents with intact cognitive function to decide on their preference, and plan their care through open discussions can ease the preparation of EOL care and ensure it reflects their desires [44]. According to a Korean national survey [2], although a significantly larger percentage of people (74.4%) perceived prepared death as a favorable death, only 20% of people were accurately aware of hospice palliative care and advanced directives. Therefore, social awareness and institutional support are required to encourage older adults and their families to receive EOL preparatory education before entering nursing homes [2].

Finally, the participants emphasized the need for legal and institutional improvements for delivering high-quality EOL care, including sufficient procurement of professional nursing manpower. This was consistent with a previous study, which highlighted the need for RNs who can understand the health conditions of older residents, recognize the changes in their health conditions, and provide emergency care [32]. A scoping review by Bolt et al. [18] also pointed out concerns that understaffing compelled task-centered rather than person-centered care, as well as cuts to nursing staff services. Therefore, it is necessary to discuss the appropriateness of nurse staffing at nursing homes and improve related systems to provide good EOL care. The legal limitation associated with the provision of palliative care at nursing homes is a big conundrum, which is discussed as a major agenda in developed countries where EOL care policies and systems have been established early [44]. The related legislation in South Korea is still at a preliminary stage since its enactment in 2016. Policy makers and health authorities should pay close attention to the difficulties experienced by RNs at nursing homes, and listen to their propositions. Furthermore, they need to examine strategies for enhancing the quality of life and comfortable death of older residents at the EOL stage, taking initiatives to improve the system.

Focusing on the experiences of RNs at nursing homes, this study did not reflect the experiences or perspectives of older residents at the EOL stage, their families, and people in other professions. In addition, the causes, conditions, and results of each experience, as well as causal relationships, were not clearly determined. As only a small number of RNs were interviewed for this study, it cannot be assumed to represent the views of all RNs in Korean nursing homes. Furthermore, the EOL care provided by participants was limited by national policies and institutional regulations; the situation in other countries would likely be different.

## 5. Conclusions

This study presented the fact that RNs at nursing homes are doing their best to care for older residents at their EOL stage and their families in a restricted environment, while facing difficulties caused by legal and institutional limitations, which often lead to defensive coping. Therefore, for these RNs to offer high-quality care, institutional improvement is critical: establishing an EOL care system at nursing homes, strengthening training processes, securing qualified nursing manpower, and reducing obstacle factors. Additionally, to provide EOL care reflecting the desire of older residents, death preparatory education, including advanced directives, needs to be socially activated so that the residents can prepare for their death in advance, while they are cognitively functional. Effective EOL care can be achieved not just by RNs, but also by the collaboration of the older residents, their family members, certified caregivers, and other professionals. Therefore, further qualitative or quantitative studies investigating the experiences and demands of EOL care from the perspectives of older residents at nursing homes, their families, and other workers will be needed.

## Figures and Tables

**Table 1:** Interview Questions.

Questions	Contents
Central question	“Tell me about your experience in caring for the older resident at the end of his or her life.”
Sub-questions	“What was the situation with the older resident at the end of his or her life?”
	“What was the caring process for the dying resident.”
	“Tell me about the process following the death of older residents.”
	“Tell me if there is a policy or procedure for EOL care at your facility.”
	“How did you communicate with the older adult and their family, colleagues, and other medical professionals?”
	“Tell me about the difference in EOL care between nursing homes and other healthcare settings.”
	“In your opinion, what facilitators or barriers exist in providing EOL care at nursing homes?”

**Table 2:** Characteristics of Participants (*n* = 11).

Participant	Gender	Age (Years)	Marital Status	Education Level	Total Clinical Career (Years)	Working Career in Nursing Homes (Years)	Position
A	Woman	27	Unmarried	Bachelor	4	0.7	Staff nurse
B	Woman	47	Married	Associate	14	2.7	Staff nurse
C	Woman	54	Married	Master	15	7	Nurse team manager
D	Woman	49	Married	Bachelor	14	10	Nurse team manager
E	Woman	48	Married	Associate	8	2	Staff nurse
F	Woman	54	Married	Master	15	10	Nurse team manager
G	Woman	50	Married	Bachelor	8	6	Nurse team manager
H	Woman	59	Unmarried	Bachelor	30	15	Director
I	Woman	55	Married	Associate	18	15	Director
J	Woman	51	Married	Associate	22	9	Staff nurse
K	Woman	49	Married	Associate	23	3.3	Director

**Table 3:** Themes and Sub-themes.

Themes	Sub-themes
Feeling fulfilled for doing my best until the last day	Fulfilling the duty for the older residents and families Feeling proud to be able to do something for the older residents at the EOL stage
Defensive coping due to legal and institutional limitations	The burden of responsibility as the sole healthcare professional in the nursing home setting Legal restrictions to nurses’ roles and limited authority Higher priority to facility operation than supporting older residents
Requirements for effective EOL Care	Building trust between the older resident’s family, nursing home employees, and on-call doctors as a buffer preventing legal issues EOL care education for nursing staffs, residents, and their families Institutional support about EOL care in nursing homes

EOL = end-of-life.

## Data Availability

Data not available due to ethical restrictions. Due to the nature of this qualitative research, participants of this study did not agree for their data to be shared publicly, so supporting data is not available.
